# CSF protein ratios with enhanced potential to reflect Alzheimer’s disease pathology and neurodegeneration

**DOI:** 10.1186/s13024-024-00705-z

**Published:** 2024-02-13

**Authors:** Sára Mravinacová, Vilma Alanko, Sofia Bergström, Claire Bridel, Yolande Pijnenburg, Göran Hagman, Miia Kivipelto, Charlotte Teunissen, Peter Nilsson, Anna Matton, Anna Månberg

**Affiliations:** 1 Division of Affinity Proteomics, Department of Protein Science, KTH Royal Institute of Technology, SciLifeLab, Stockholm, Sweden; 2 Division of Clinical Geriatrics, Department of Neurobiology, Care Sciences and Society, Karolinska Institutet, Stockholm, Sweden; 3 Division of Neurogeriatrics, Department of Neurobiology, Care Sciences and Society, Karolinska Institutet, Stockholm, Sweden; 4 Neurochemistry Lab, Department of Laboratory Medicine, Amsterdam Neuroscience, Amsterdam UMC, Vrije Universiteit, Amsterdam, Netherlands; 5 Department of Neurology, Alzheimer Centre, Amsterdam UMC, Vrije Universiteit, Amsterdam, Netherlands; 6 Ageing Epidemiology (AGE) Research Unit, Imperial College London, London, United Kingdom; 7 Theme Inflammation and Aging, Karolinska University Hospital, Stockholm, Sweden; 8 Institute of Public Health and Clinical Nutrition, University of Eastern Finland, Kuopio, Finland

**Keywords:** Alzheimer’s disease, CSF, Neurodegeneration, Cognitive decline, Protein ratios, Protein profiling, Affinity proteomics, Inter-individual variability

## Abstract

**Background:**

Amyloid and tau aggregates are considered to cause neurodegeneration and consequently cognitive decline in individuals with Alzheimer’s disease (AD). Here, we explore the potential of cerebrospinal fluid (CSF) proteins to reflect AD pathology and cognitive decline, aiming to identify potential biomarkers for monitoring outcomes of disease-modifying therapies targeting these aggregates.

**Method:**

We used a multiplex antibody-based suspension bead array to measure the levels of 49 proteins in CSF from the Swedish GEDOC memory clinic cohort at the Karolinska University Hospital. The cohort comprised 148 amyloid- and tau-negative individuals (A-T-) and 65 amyloid- and tau-positive individuals (A+T+). An independent sample set of 26 A-T- and 26 A+T+ individuals from the Amsterdam Dementia Cohort was used for validation. The measured proteins were clustered based on their correlation to CSF amyloid beta peptides, tau and NfL levels. Further, we used support vector machine modelling to identify protein pairs, matched based on their cluster origin, that reflect AD pathology and cognitive decline with improved performance compared to single proteins.

**Results:**

The protein-clustering revealed 11 proteins strongly correlated to t-tau and p-tau (tau-associated group), including mainly synaptic proteins previously found elevated in AD such as NRGN, GAP43 and SNCB. Another 16 proteins showed predominant correlation with Aβ42 (amyloid-associated group), including PTPRN2, NCAN and CHL1. Support vector machine modelling revealed that proteins from the two groups combined in pairs discriminated A-T- from A+T+ individuals with higher accuracy compared to single proteins, as well as compared to protein pairs composed of proteins originating from the same group. Moreover, combining the proteins from different groups in ratios (tau-associated protein/amyloid-associated protein) significantly increased their correlation to cognitive decline measured with cognitive scores. The results were validated in an independent cohort.

**Conclusions:**

Combining brain-derived proteins in pairs largely enhanced their capacity to discriminate between AD pathology-affected and unaffected individuals and increased their correlation to cognitive decline, potentially due to adjustment of inter-individual variability. With these results, we highlight the potential of protein pairs to monitor neurodegeneration and thereby possibly the efficacy of AD disease-modifying therapies.

**Supplementary Information:**

The online version contains supplementary material available at 10.1186/s13024-024-00705-z.

## Introduction

Alzheimer’s disease (AD) is a neurodegenerative disorder with a long asymptomatic phase that affects millions of individuals worldwide, mostly those aged over 65 years [1]. The pathology of AD is characterized by the accumulation of two types of aggregates in the brain, amyloid beta (Aβ) plaques and tau tangles. These aggregates are believed to cause neurodegeneration and consequently lead to cognitive decline and memory loss. Large efforts have been made to develop treatments targeting these aggregates and/or their preceding building blocks, with the ambition to slow down disease progression. The US Food and Drug Administration (FDA) recently granted approval for two antibody-based therapies targeting amyloid plaques. Aducanumab [2] received accelerated approval, and lecanemab [3] full approval by the authority. However, no disease-modifying therapies have been approved by the European Medicine Authority to this date. Additionally, several therapies targeting either amyloid or tau are being evaluated in various stages of clinical trials [4].

With new treatment possibilities emerging, development of biomarkers to monitor treatment efficacy is of great importance. Amyloid and tau positron emission tomography (PET) scans, as well as cerebrospinal fluid (CSF) biomarkers such as Aβ42, phosphorylated tau (p-tau) and total tau (t-tau) are used to select participants in AD trials, to evaluate target engagement, as well as to evaluate the disease-modifying effect reflected by the changes in AD pathology[5]. However, additional biomarkers providing complementary assessment to amyloid and tau could enable further evaluation of the effect of the treatment on a molecular level. Neuronal loss measured by cortical thickness on magnetic resonance imaging together with fludeoxyglucose (FDG) PET, measuring synaptic loss through a decrease in metabolic activity, can be used to estimate neurodegeneration [5–7]. However, the analysis and interpretation of these images are cumbersome. Moreover, the PET scanning procedure is costly and not widely available.

An alternative approach for assessing neurodegeneration is to measure synaptic damage through levels of synaptic proteins in CSF. Synaptic damage has been widely recognized as an indicator of neurodegeneration and stands as the most prominent predictor of cognitive decline in AD [8, 9]. Neurogranin (NRGN) is a promising fluid biomarker for synaptic damage, showing both high CSF levels in AD and correlation with cognitive decline [10–14], as well as correlation with hippocampal volume and brain metabolic activity measured by FDG PET [11]. Furthermore, CSF NRGN has previously been included as an outcome measure in the clinical trial for lecanemab [3]. Several other synaptic proteins have also been shown as strongly associated with AD, including neuromodulin (GAP43) [12, 15–18] and beta synuclein (SNCB) [15, 19]. All these proteins were reported to highly correlate with CSF p-tau and t-tau levels [10, 11, 13–15, 19], suggesting a close relationship between tau pathology and synaptic damage.

In our previous study, we investigated the association between 104 CSF proteins and CSF Aβ42 and tau levels in cognitively unimpaired 70-year-old individuals [20]. We demonstrated that a subset of these proteins correlated with CSF t-tau, p-tau and Aβ42 levels, many of which were of synaptic origin. Interestingly, the strength of the correlations differed in amyloid-positive and amyloid-negative individuals, suggesting different associations of CSF proteins, including synaptic proteins, to amyloid and tau pathology already in the early stages of the disease. Here, we extend our investigation to a memory clinic cohort comprised of individuals with subjective cognitive decline and individuals in different stages of the AD continuum. Within these, we focus on amyloid and tau negative (A-T-) and amyloid and tau positive (A+T+) individuals, categorised based on CSF Aβ42/40 ratio and p-tau levels. Moreover, we explore the combination of CSF proteins reflecting synaptic damage such as GAP43, NRGN, SNCB and amphiphysin (AMPH) in pairs with other brain-derived proteins to evaluate their combined ability to reflect disease pathology and hence their potential to be used in clinical trials to assess the efficacy of new AD therapies.

## Materials and methods

### Sample collection

The discovery cohort included patients from the Karolinska University Hospital Medical Unit Aging Memory clinic (GEDOC database and biobank) in Solna, examined between 2019–2021. The cohort consisted of 241 individuals diagnosed with probable Alzheimer’s disease (*n* = 44), mild cognitive impairment (*n* = 65) or subjective cognitive decline (SCD) (*n* = 132), according to the national guidelines of Sweden that have been established by the Swedish Board of Health and Welfare [21]. The diagnostic examination process has been described in detail previously [22]. Patients remitted to the memory clinic underwent extensive examinations that encompass, for instance, clinical examinations, neuropsychological assessments, blood chemistry analyses, CSF biomarker measurements and MRI. The diagnosis of each patient was evaluated and set by a multidisciplinary team. The extensive cognitive examinations included: Mini Mental State Examination (MMSE), Montreal Cognitive Assessment (MoCA) total points, Rey Auditory Verbal Learning Test (RAVLT) learning, Rey Complex Figure memory (RCF), Digit Symbol-Coding (WAIS-IV) (KOD).

The samples included in the validation cohort were selected from the Amsterdam Dementia Cohort, and consisted of 26 probable AD patients and 26 SCD patients. All patients underwent extensive dementia screening at baseline, including physical and neurological examination, EEG, MRI and laboratory tests. Neuropsychological assessments were performed and included the MMSE for global cognition. Diagnoses were made by consensus in a multidisciplinary meeting. Probable AD was diagnosed according to the core clinical National Institute on Aging–Alzheimer's Association (NIA-AA) criteria. All AD patients were A+T+ in CSF. Diagnosis of SCD was determined when the results of all clinical examinations were normal, and there was no psychiatric diagnosis. All SCD patients were A-T- in CSF.

### Sample classification

To explore the association between the measured proteins and amyloid and tau pathology in the discovery cohort, the individuals were classified based on CSF Aβ42/40 ratio and CSF p-tau levels. Individuals with Aβ42/40 ratio × 10 < 0.68 were classified as amyloid positive and those with p-tau levels ≥ 58 pg/ml as tau positive. Based on their combined amyloid and tau status, the individuals were divided into four groups: A-T- (*n* = 148), A-T+ (*n* = 9), A+T- (*n* = 19) and A+T+ (*n* = 65) (Supp. Fig. 1). Only A-T- and A+T+ individuals were further included in this study. A-T+ and A+T- individuals were excluded due to insufficient sample representation. For analysis, all individuals with probable AD, MCI, or SCD were included in the A+T+ group (*n* = 65). However, only patients with SCD were included in the A-T- group (*n* = 106) (Table 1), unless otherwise stated.

**Table 1:** Sample demographics

	Discovery cohort		Validation cohort	
	A-T- with SCD	A+T+	A-T- with SCD	A+T+
Number of individuals	106	65	26	26
Diagnosis - SCD/MCI/AD	106/0/0	11/19/35	26/0/0	0/0/26
Age^a,b^	59 (51 –69)	62 (51–74)	59 (51–68)	60 (51–68)
Sex - F/M^c^	65/41	41/24	6/20	17/9
Aβ42 (pg/ml)^a^	1160 (562–2000)	540 (248–990)	1106 (867–1495)	636 (274–804)
Aβ40 (pg/ml) ÷ 10 ^a^	1134 (542–1923)	1225 (558–2185)	-	-
Aβ42/Aβ40 × 10^a^	1.01 (0.73–1.29)	0.47 (0.26–0.67)	-	-
t-tau (pg/ml)^a^	214 (75–907)	538 (324–1210)	232 (88–349)	784 (413–2160)
p-tau (pg/ml)^a^	32 (14–56)	82 (57–190)	38 (22–50)	94 (64–185)
NfL (pg/ml) ^a,d^	670 (310–3960)	1,130 (650–6,550)	-	-
Albumin CSF/serum ratio^e^	5.70 (2.50–14.40)	5.4 (2.6–11.1)	-	-
MMSE ^a,f^	28.0 (16.0–30.0)	23.0 (14.0–30.0)	29 (25–30)	22 (6–28)
MoCA ^a,f^	26.0 (13–30.0)	20.0 (5.0–28.0)	-	-
KOD ^a,f^	56 (23–98)	44 (1–72)	-	-
RAVLT ^a,f^	50 (27–69)	31 (3–64)	-	-
RCF ^a,f^	18 (2–32)	9 (0–33)	-	-

^a^The data is presented in the format: median (range)

^b^Significant difference in age distribution was observed between A-T- with SCD and A+T+ groups in the discovery cohort (*p* = 1.66×10^–4^, Wilcoxon test). No significant difference was observed in the validation cohort (*p* = 0.99)

^c^No significant difference in sex distribution was observed between A-T-with SCD and A+T+ groups in the discovery cohort (*p* = 0.87, Fisher’s exact test), but a significant difference was observed in the validation cohort (*p* = 0.005)

^d^NfL data availability in discovery cohort [N]: A-T- with SCD: 102, A+T+:64

^e^Albumin CSF/serum ratio data availability [N]: A-T- with SCD: 96, A+T+: 62

^f^Cognitive score availability in discovery cohort [N]: A-T- with SCD: 74 MMSE, 92 MoCA, 66 KOD, 70 RAVLT, 69 RCF; A+T+: 44 MMSE, 53 MoCA, 34 KOD, 41 RAVLT, 36 RCF. Cognitive score availability in validation cohort [N]: A-T-: 25 MMSE, A+T+: 26 MMSE

To validate the findings, the external validation cohort was classified in a similar manner. However, Aβ42 levels were used instead of Aβ42/40 ratio to determine the presence of amyloid pathology, as Aβ40 levels were not available for this cohort. Here, samples with Aβ42 levels < 813 pg/ml were classified as amyloid positive and with p-tau levels > 55 pg/ml as tau positive [23]. Final classification in the validation cohort based on the combined amyloid and tau status resulted in two groups, A-T- (*n* = 26) and A+T+ (*n* = 26) (Table 1, Supp. Fig. 1). These groups were identical to the diagnostic groups (Healthy controls and AD, respectively) as the AT status was used to determine the diagnosis.

### Protein analysis with suspension bead array

A multiplex antibody-based suspension bead array was used to measure the levels of 73 proteins, pre-selected based on previous published and in-house unpublished neuroproteomic studies, complemented with targets from literature. Each antibody was immobilized onto the surface of color-coded magnetic beads (MagPlex, Luminex corp.) using NHS-EDC chemistry, as described previously [24]. The beads were subsequently pooled to form a multiplex bead array. All antibodies used in this study were polyclonal rabbit antibodies produced within the Human Protein Atlas (www.proteinatlas.org), except for the angiotensinogen (AGT) antibody (AF3156-SP, R&D Systems).

The CSF samples were transferred into 96-well PCR plates in a stratified randomisation manner based on diagnosis, age and sex. Next, the crude samples (1/2 dilution) were directly labelled with an approximated tenfold molar excess of biotin (NHS-PEG4-biotin, A39259, ThermoFisher Scientific), as previously described [25].The labelled samples were further diluted to a final dilution of 1/25 and heat-treated for 30 min at 56 °C before incubation with the prepared bead array at room temperature overnight. After washing the unbound proteins, the antibody-bound protein targets were labelled with a streptavidin-bound fluorophore and quantified using the Flexmap 3D instrument (Luminex corp.). Data was acquired as a median fluorescent intensity per bead ID and per sample (relative quantification).

### CSF amyloid, tau, NfL and CSF and serum albumin measurements

For samples in the discovery cohort analysed before August 22, 2019 and for all samples in the validation cohort, AD biomarkers Aβ40 (only in discovery cohort), Aβ42, t-tau and p-tau were measured in CSF by commercially available ELISAs (Innotest AMYLOID (1– 40), Innotest AMYLOID (1– 42), Innotest hTAU-Ag and Innotest Phosphotau (181P); Fujirebio), following the manufacturer’s instructions. Samples in the discovery cohort analyzed after August 22, 2019 were measured using the Lumipulse G-series chemiluminescent enzyme immunoassay (Fujirebio Europe). NfL measurements were performed using a commercial ELISA (Uman Diagnostics, 10-7001). CSF and serum albumin concentrations were measured using the BN ProSpec/Atellica NEPH platform (Siemens Healthineers). All analyses in the discovery cohort were performed at the Karolinska University Hospital Laboratory, and in the validation cohort at the Neurochemistry Laboratory, Amsterdam UMC.

### Data analysis and visualizations

Data processing, analysis and visualizations were performed using the open-source R statistical software (4.2.2) with extra packages vroom, tidyverse, ggpubr, ggbeeswarm, ggrepel, pheatmap, stats, scales, and patchwork. Additional packages and functions used in this study are stated in the respective data analysis sections. The figures were further adjusted for clarity (e.g., figure legends) using the vector graphic editor Affinity Designer (1.8.6) (Serif, West Bridgford, UK).

#### Data adjustment and quality control

The raw data generated in the multiplex protein profiling were adjusted for technical variations in two steps. First, the data were adjusted to minimize the effect of delayed instrument readout. For this, a robust linear model (rlm, MASS) was constructed for each protein where the response variable was the protein fluorescent intensity and the predictor the sample position in the plate. The model residuals were thereafter added to the median protein signal intensity to obtain the adjusted values for each protein and sample. The data was further adjusted for potential differences between the sample plates using the MA-individual normalization [26]. To evaluate the technical variation of each protein assay, three sample pool replicates were included in each sample plate to assess intra-assay reproducibility. Data adjustment steps were followed by further quality control based on inter-assay correlation (required Spearman *rho* ≥ 0.7) and background evaluation. Finally, data analysis was conducted on 49 proteins (Supp. Table 2), which had a median intra-assay CV of 3.5% with the range of 1.1–10.9% (with only one protein, CHIT1, having CV over 10%).

#### Univariate analysis

The differences in CSF levels of the proteins measured with the suspension bead array between A-T- individuals with SCD and A+T+ individuals were tested using the non-parametric Wilcoxon rank-sum two-sided test (wilcox.test, stats). The obtained *p*-values were adjusted for false discovery rate (FDR) using the Benjamini-Hochberg correction for multiple hypothesis testing. Proteins with adjusted *p*-value < 0.05 were considered significantly different between the tested groups. The same statistical approach was used to compare ROC AUC values between protein pairs from different clusters in both discovery and validation cohorts, and to compare GAP43/PTPRN2 ratio, SNCB/PTPRN2 ratio, GAP43 and PTPRN2 between the different diagnostic groups in A-T- and A+T+ individuals in the discovery cohort, without *p*-value adjustment.

#### Protein clustering

Correlation between the measured proteins and CSF AD markers (Aβ40, Aβ42, Aβ42/40, t-tau, p-tau, NfL) was calculated using Spearman correlation (cor, stats). Hierarchical clustering (hclust, stats) was performed to cluster the measured proteins based on their correlation to CSF AD markers. Euclidean distance was used as the similarity measure and Ward’s method (ward.D2) was used for the clustering. To visualize the clustering result, a heatmap was created (pheatmap) using the same clustering method. The heatmap was further annotated with correlations (Spearman) between the individual protein levels and albumin quotient. The same approach was used to cluster proteins based on their correlation to each other for both A-T- and A+T+ individuals, and further to visualise correlation between cognitive scores and CSF AD markers amyloid beta peptides, tau and NfL.

The network graph visualisation of correlations between all measured proteins in the A-T- with SCD and A+T+ individuals were generated using tidygraph and ggraph R packages. Only correlations with |rho| > 0.5 were included in the graph which was created using the Fruchterman-Reingold layout algorithm.

#### Support vector machine modelling

Support vector machine modelling (SVM) was used to assess the capability of CSF protein pairs to differentiate A+T+ from A-T- (SCD) individuals. Separate models were created to test all possible protein pairs within and between the amyloid- and tau-associated clusters. The SVM models were constructed using a training sample set comprising 70% of the discovery cohort samples, and each model was evaluated using the remaining 30% of samples from the same cohort. To counter group size biases, the A-T- sample group was randomly undersampled to the size of the A+T+ group (*n* = 65) prior to the split. The split into training and test sets was repeated 101 times with different seeds resulting in 101 models per protein pair, with the same seed used for undersampling. To optimise the models the “cost” parameter was tuned in the training part with 10 times cross-validation, with the tested values of 0.1, 1, 10 and 100. The linear kernel was applied to all models and the protein data underwent log transformation, scaling, and centering to the median (scale, baseR with colMedians, matrixStats). Model performance was compared using the receiver operating characteristic analysis (ROC) area under the curve (AUC) (roc, pROC). The confidence interval for the median model AUC was estimated using the bootstrap resampling with 1000 iterations (ci, pROC). The same modelling procedure was used to construct SVM models with albumin CSF/serum ratio included as a predictor variable, and for single proteins from the tau-associated cluster as single predictor variables (GAP43, SNCB, NRGN and AMPH).

To validate the robustness of the modelling results, we replicated the model with the median AUC from the 101 constructed models for each protein pair in the independent validation cohort. The sample sizes of the diagnostic groups within this cohort were equal, allowing for passing all samples into the model. The resulting AUC with confidence interval for each protein pair was recorded and evaluated.

#### Cognitive data correlation analysis

The correlation between the single protein or protein pair ratios and cognitive scores was calculated using the Pearson correlation (cor, stats). All individuals with cognitive score data available within the A-T- individuals with SCD and A+T+ individuals were passed to the correlation (Discovery cohort – MMSE: *n* = 118, MoCA: *n* = 145, KOD: *n* = 100, RAVLT: *n* = 111, RCF: *n* = 105; Validation cohort – MMSE: *n* = 51). The statistical comparison of correlations between the protein pair ratios from different clusters or between the tau-associated protein/amyloid-associated protein ratios and single proteins from amyloid- and tau-associated clusters were calculated using the Wilcoxon rank-sum two-sided test (wilcox.test, stats) using the absolute correlation values.

## Results

In this study, we measured the levels of 49 pre-selected proteins in CSF from A-T- and A+T+ individuals from the Swedish memory clinic cohort, and an independent Amsterdam Dementia Cohort. We aimed to identify candidate biomarkers or pairs of biomarkers that reflect disease pathology and cognitive decline.

### Brain-derived proteins associate differently with CSF amyloid and tau

**Fig. 1:**
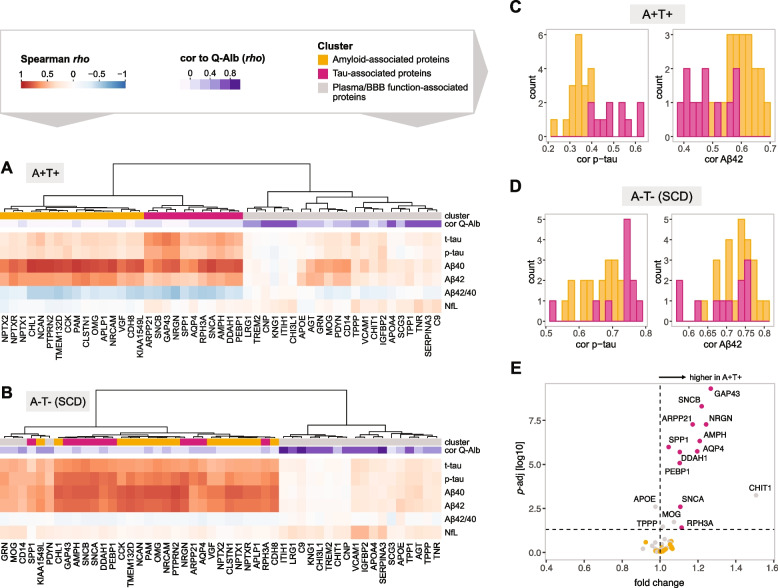
Correlation of suspension bead array-measured CSF proteins to amyloid beta peptides, tau and NfL markers. **A** Correlation heatmap for amyloid and tau positive (A+T+) individuals with SCD, MCI or AD. The heatmap is annotated with Spearman’s correlations of the proteins to albumin CSF/serum quotient (Q-Alb) **B** Correlation heatmap for amyloid and tau negative (A-T-) individuals with SCD, annotated with clusters from (A) and Spearman’s correlation with Q-Alb (**C**, **D**) Stacked histogram showing the correlation coefficients of amyloid-associated and tau-associated cluster proteins with p-tau (left) and Aβ42 (right) in A+T+ individuals (C) and A-T- (SCD) individuals (D). (**E**) Volcano plot showing CSF proteins with altered levels in A-T- (SCD) individuals compared to A+T+ individuals. The proteins are colored based on their cluster origin

First, we investigated the correlation between the levels of the 49 pre-selected proteins in CSF and CSF levels of t-tau, p-tau, Aβ40, Aβ42, Aβ42/40 ratio and NfL in A+T+ individuals with a diagnosis of probable AD, MCI, or SCD (*n*=65) and A-T- individuals with SCD (*n*=106). In A+T+ individuals, the analysis revealed two main protein clusters (Fig. 1A). The first cluster (grey) comprised 22 proteins, which showed no or weak correlation with AD markers. The proteins did however display strong correlations with the albumin CSF/serum quotient, suggesting that they originate from non-central nervous system (CNS) tissues, have a mixed origin in the CNS and periphery, or have association to the brain barrier function. In contrast, proteins in the second cluster (orange+pink, *n* = 27) showed strong correlation with AD markers but weak associations with the albumin quotient. Transcriptomic data from the Human Protein Atlas (v. 23) [27] shows that 22 out of the 27 proteins in the second cluster have elevated expression of the corresponding genes in the CNS tissues compared to other tissues (Supp. Table 2), further supporting their predominantly CNS origin. A similar clustering pattern was observed in A-T- individuals, where the majority of the proteins clustered in the same two groups (Fig. 1B). In the A+T+ individuals, the second cluster was further divided into two subclusters based on protein correlation with amyloid beta peptides and tau. The first subcluster (pink) comprised eleven proteins with increased correlation with t-tau and p-tau (further referred to as the tau-associated cluster). The second subcluster (orange) consisted of 16 proteins that showed a weaker correlation with p-tau and t-tau but a stronger correlation with Aβ42 (further referred to as the amyloid-associated cluster) (Fig. 1A, C, Supp. Fig. 2). Notably, subclustering was not observed in A-T- individuals, where 25 out of the 27 proteins clustered together but showed more similar correlations with amyloid beta peptides and tau CSF markers (Fig. 1B, D, Supp. Fig. 3). To further investigate our findings, we performed the Wilcoxon rank-sum test for each protein between the A+T+ and A-T- groups. All eleven proteins in the tau-associated cluster showed significant increase in the A+T+ group, with GAP43 being the most significantly increased protein (adjusted *p*-value = 5×10^-10^) (Fig. 1E, Supp. Fig. 4, Supp. Table 3). Notably, none of the 16 proteins in the amyloid-associated cluster showed significantly different CSF levels between the groups (Fig. 1E, Supp. Fig. 2). Despite this, the levels of the majority of proteins in both clusters showed positive correlations with each other (median Spearman *rho* in A+T+ = 0.83; A-T- SCD = 0.82) (Supp. Figs. 2, 3, 5 and 6). Notably, correlations between the amyloid- and tau-associated proteins and NfL were generally weak in both the studied sample groups (Fig. 1A, B). In A-T- individuals, all proteins showed weak positive correlation with NfL. In A+T+ individuals, amyloid-associated proteins showed no or slightly negative correlations with NfL, while weak positive correlations were observed for tau-associated proteins, with SNCB being the most correlated protein (*rho* = 0.25).

### Protein pairs from different clusters predict amyloid and tau status with high accuracy

**Fig. 2:**
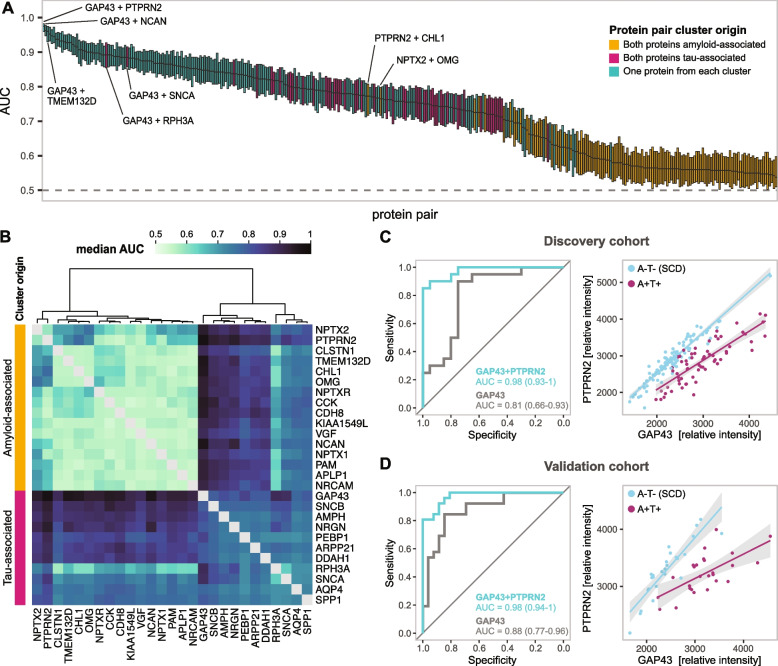
Performance of protein pairs in distinguishing A+T+ and A-T- individuals. **A** SVM modelling results showed as boxplots ordered based on median ROC AUC. In the modelling, levels of the two CSF proteins were used as the predictor variables and the amyloid and tau status as a response variable, with only SCD individuals included in the A-T- sample group. Each boxplot corresponds to one protein pair and includes the results from 101 repeated models. Boxplot whiskers and outlying data points were removed for visualisation purposes. **B** A heatmap of the median ROC AUC values from SVM models from each protein pair. The heatmap is clustered based on the ROC AUC results and annotated on the left with clustering results from Fig. 1A, based on the correlation of the individual proteins to amyloid beta peptides, tau and NfL markers in A+T+ individuals. **C**, **D** ROC curves constructed from the best protein pair (GAP43 and PTPRN2) median model predictions for discovery cohort (C) and validation cohort (D) (left). The relationship between the CSF levels of GAP43 and PTPRN2 showed as scatterplots with datapoints (individual samples) colored based on amyloid and tau status in discovery (C) and validation (D) cohort (right). The relationship is further visualized using robust linear regression

Based on the different associations of the CNS-derived proteins with amyloid beta peptides and tau CSF levels in the A+T+ individuals, we hypothesised that combining proteins from the tau- and amyloid-associated clusters may increase their potential to discriminate A+T+ individuals from A-T- individuals. To test this hypothesis, we assembled all protein pair combinations both between and within the two clusters and evaluated their predictive performance using support vector machine modelling. CSF levels of the two proteins in the pair were used as predictor variables and the amyloid and tau status as the outcome variable. Protein pairs combined from the two clusters achieved significantly higher ROC AUCs compared to protein pairs with both components originating from the same cluster, whether amyloid- or tau-associated (*p*-value = 1×10^–45^ and 1×10^–8^, respectively) (Fig. 2A-B, Supp. Fig. 7). This was followed by protein pairs with both components originating from the tau-associated cluster (*p*-value = 8×10^–25^ for comparison with amyloid-associated protein pairs). Protein pairs with both components from the amyloid-associated cluster demonstrated the lowest predictive accuracy. The protein pair with the best predictive performance was GAP43 + protein tyrosine phosphatase receptor type N2 (PTPRN2) (median AUC = 0.98 (CI 0.93–1)) (Fig. 2C), followed by GAP43 + neurocan (NCAN) (median AUC = 0.96 (CI 0.9-1) (Table 2). Importantly, neither sex nor age seemed to affect the separation between A-T- and A+T+ individuals provided by the protein pairs. On the other hand, the separation clearly reflected the amyloid and tau pathology represented by CSF Aβ42/40 ratio and CSF p-tau (Supp. Fig. 8). In total, 19 protein pairs reached median AUC higher than 0.90 and all of these pairs had one protein originating from each protein cluster. These 19 pairs were composed of a combination of four proteins from the tau-associated cluster, namely GAP43, NRGN, SNCB and AMPH, and 14 proteins from the amyloid-associated cluster, including PTPRN2, NCAN, transmembrane protein 132D (TMEM132D), and neural cell adhesion molecule L1-like protein (CHL1) (Table 2). We also investigated whether including albumin CSF/serum ratio as a predictor variable together with the 19 best-performing protein pairs could further increase the prediction capacity. However, no increase in performance was observed (Supp. Table 4).

**Table 2:** ROC AUC for protein pairs in the discovery cohort, and corresponding validation cohort data

Tau-associated protein	Amyloid-associated protein	AUC (CI) discovery	AUC (CI) validation
GAP43	PTPRN2	0.98 (0.93–1)	0.98 (0.94–1)
GAP43	NCAN	0.96 (0.9–1)	0.94 (0.86–1)
GAP43	TMEM132D	0.96 (0.89–1)	0.99 (0.98–1)
GAP43	CHL1	0.95 (0.88–1)	0.97 (0.92–1)
GAP43	CCK	0.95 (0.87–1)	0.94 (0.87–0.99)
GAP43	NPTX2	0.94 (0.84–1)	0.97 (0.91–1)
GAP43	CDH8	0.94 (0.84–1)	0.98 (0.92–1)
NRGN	PTPRN2	0.94 (0.83–1)	0.94 (0.87–0.99)
SNCB	PTPRN2	0.93 (0.84–1)	1 (0.98–1)
GAP43	PAM	0.93 (0.84–0.99)	0.98 (0.95–1)
SNCB	CCK	0.93 (0.84–0.99)	0.95 (0.89–1)
GAP43	OMG	0.93 (0.84–0.99)	0.91 (0.83–0.98)
GAP43	APLP1	0.93 (0.84–0.99)	0.89 (0.79–0.96)
GAP43	NPTXR	0.93 (0.84–1)	0.87 (0.75–0.97)
AMPH	PTPRN2	0.92 (0.81–0.99)	0.93 (0.86–0.98)
GAP43	NRCAM	0.92 (0.8–0.99)	0.9 (0.81–0.97)
NRGN	NCAN	0.92 (0.83–0.99)	0.9 (0.8–0.98)
GAP43	NPTX1	0.91 (0.8–0.99)	0.9 (0.8–0.97)
GAP43	KIAA1549L	0.91 (0.8–1)	0.88 (0.76–0.97)

Next, we evaluated whether the protein pairs showed better discrimination power between the sample groups compared to the four individual proteins from the tau-associated cluster alone. For this, we repeated the modelling analysis with GAP43, NRGN, SNCB, or AMPH CSF levels as the only predictor variable. This approach showed considerably lower performance, with the highest median AUC of 0.81 (CI 0.66–0.93) for GAP43 (Fig. 2C), followed by SNCB, NRGN and AMPH (median AUC of 0.78 (CI 0.62–0.91), 0.76 (CI 0.58–0.9) and 0.75 (CI 0.58–0.9), respectively). This highlights the added value of protein pairs in discriminating individuals with and without AD pathology.

### Protein pair predictions were reproduced in an independent cohort

To validate the performance of the CSF protein pairs, we applied the model with median AUC derived from the discovery cohort for each protein pair to an independent validation cohort (26 A+T+ and 26 A-T- individuals). The results confirmed our previous findings, again showing the best separation for protein pairs combined from the tau- and amyloid-associated clusters compared to other protein combinations (Supp. Figs. 9, 10, 11). Thirteen out of the 19 protein pairs that demonstrated an AUC higher than 0.90 in the discovery cohort reached AUC over 0.9 also in the validation cohort. The remaining six pairs reached an AUC of 0.9 or slightly below (Table 2). Notably, GAP43+PTPRN2 maintained equally high performance as in the discovery cohort, with AUC = 0.98 (CI 0.94–1) and again demonstrated a better separation compared to GAP43 alone (AUC = 0.88 (CI0.77–0.96)) (Fig. 2D). The protein pair with the best performance in the validation cohort was SNCB+PTPRN2 (AUC = 1).

### Protein pair ratios correlate negatively with cognitive decline

**Fig. 3:**
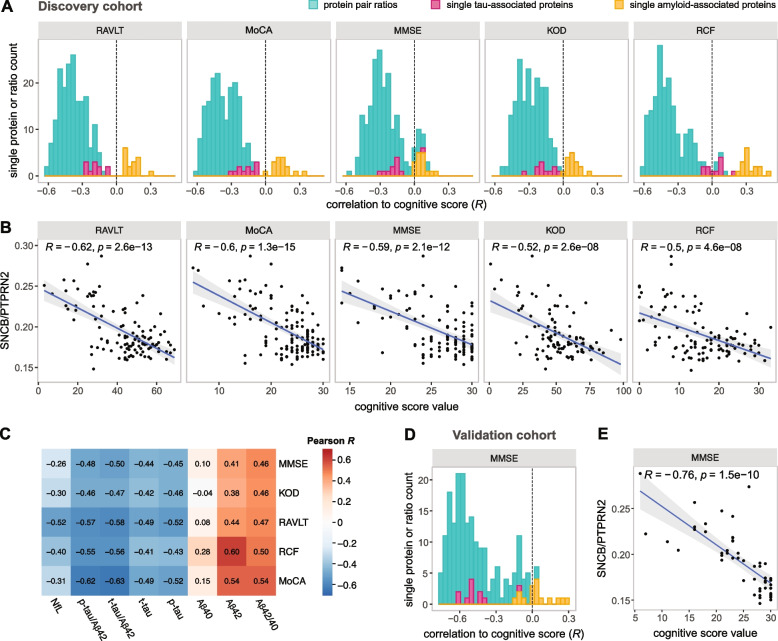
Correlation between CSF protein levels and cognitive scores. **A** Stacked histogram showing correlations coefficients between CSF proteomic data and cognitive data in the discovery cohort. The correlations were calculated and shown for ratios of CSF levels of two proteins from distinct clusters, and for CSF levels of individual proteins. **B** Correlation between CSF SNCB/PTPRN2 ratio and individual cognitive scores in the discovery cohort. One data point was removed for visualisation reasons from the MoCA correlation (MoCA = 13, SNCB/PTPRN2 = 0.36). The trendline was obtained using linear regression. **C** Correlation between CSF amyloid beta peptides, tau and NfL markers and cognitive scores in the discovery cohort. **D** Stacked histogram showing correlations between CSF proteomic data and cognitive data in the validation cohort. The correlations were calculated and shown for ratios of CSF levels of two proteins from distinct clusters, and for CSF levels of individual proteins. **E** Correlation between CSF SNCB/PTPRN2 ratio and MMSE in the validation cohort

To evaluate the association of the protein pairs with cognitive decline, we combined the CSF levels of protein pairs with one protein from the tau-associated and one from the amyloid-associated cluster by computing the ratio (tau-associated/amyloid-associated). To allow for comparison, we further calculated ratios between proteins originating from the same cluster (with the proteins randomly positioned in the ratio). The resulting ratios with one protein from each cluster in the discovery cohort were negatively correlated to all five cognitive scores (RAVLT, MoCA, MMSE, KOD, RCF), and showed stronger absolute correlations compared to protein ratios between proteins originating in the same cluster, whether amyloid- or tau-associated (Supp. Fig. 12). Moreover, these correlations were significantly stronger compared to absolute correlations of single proteins for all the five cognitive scores (Fig. 3A, Supp. Fig. 13). Notably, while individual proteins associated with tau showed negative correlations to the cognitive scores, proteins associated with amyloid generally showed positive correlations (Fig. 3A). Single tau-associated proteins had stronger absolute correlations than single amyloid-associated proteins with all the cognitive scores beside RCF, where amyloid-associated proteins reached stronger correlations. Among the protein ratios, SNCB/PTPRN2 displayed the strongest negative correlation with four out of five cognitive scores (*R* for RAVLT = –0.62, MoCA = –0.61, MMSE = –0.59 and KOD = –0.52) (Fig. 3B). The ARPP21/NPTXR ratio had the strongest correlation to RCF (*R* = –0.59), although several other protein pairs showed similar correlations (Supp. Table 5). These correlations were similar to correlations of t-tau/Aβ42 and p-tau/Aβ42 ratios (strongest with MoCA, *R* = –0.63 and *R* = –0.62, respectively) (Fig. 3C). Furthermore, the correlations between protein ratios and cognitive scores were comparable to the correlations between the cognitive scores themselves (Supp. Fig. 14).

In the validation cohort, the protein ratios with one protein from each cluster demonstrated significantly stronger correlations to MMSE compared to protein ratios between proteins originating in the same cluster (Supp. Fig. 12), as well as compared to single amyloid-associated proteins, but did not reach statistically stronger absolute correlations compared to single tau-associated proteins (Fig. 3D, Supp. Fig. 13). However, fourteen out of fifteen most correlated ratios to MMSE in the discovery cohort (presented in Supp. Table 5) reached stronger correlations compared to the most correlated single tau-associated protein in the validation cohort (GAP43, *R* = –0.62), suggesting consistency in the data. Among the protein ratios in the validation cohort, the GAP43/PTPRN2 ratio showed the strongest correlation (*R* = –0.77). Notably, the SNCB/PTPRN2 ratio showed the second highest correlation (*R* = –0.76) (Fig. 3E). Several other ratios also displayed correlations in a similar range (Supp. Table 6).

### Protein pairs identify AD-diagnosed individuals with negative AT status

**Fig. 4:**
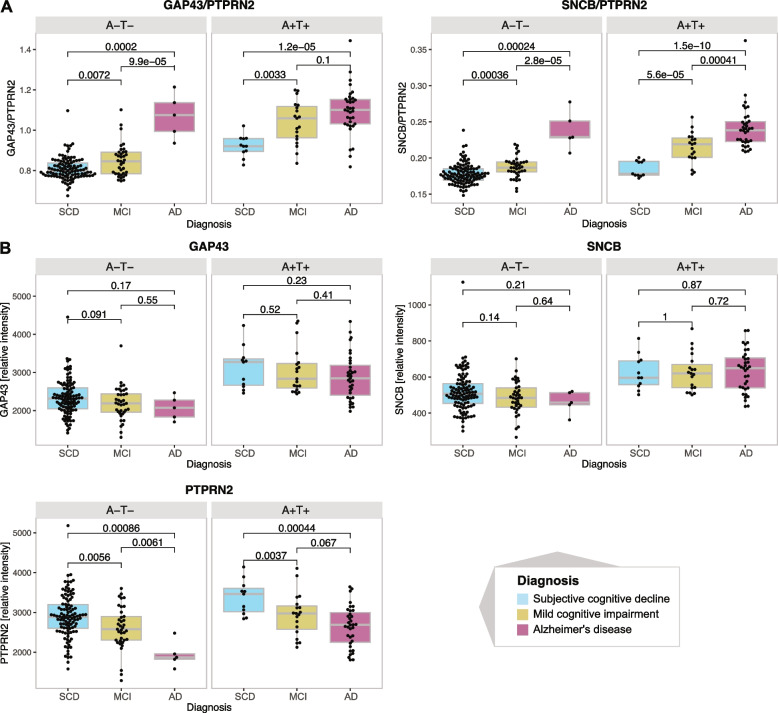
Protein ratio and individual protein CSF level distribution in individuals stratified based on AT status and diagnosis. **A** GAP43/PTPRN2 and SNCB/GAP43 distribution in the discovery cohort, **B** GAP43, SNCB and PTPRN2 levels in the discovery cohort. Distributions between the individual groups are compared using the Wilcoxon rank-sum test and the resulting *p*-values are reported in the individual plots

Furthermore, we investigated the GAP43/PTPRN2 and SNCB/PTPRN2 ratios in the full discovery cohort (*n*= 213) with respect to the clinical diagnosis of patients, including both AD and MCI patients in the A-T- group (total *n*= 148). As expected, we observed that the A+T+ individuals generally exhibited higher ratios compared to A-T- individuals (Fig. 4A). Interestingly, the protein ratios also reflected the degree of cognitive impairment within both the A-T- and A+T+ groups. For example, both GAP43/PTPRN2 and SNCB/PTPRN2 ratios clearly distinguished probable AD individuals with negative AT status from those diagnosed with SCD or MCI. This trend was not observed when considering only GAP43 or SNCB CSF levels independently (Fig. 4B). However, PTPRN2 CSF levels showed a clear decrease with the level of impairment in both AT groups.

## Discussion

In this study, we assessed the ability of CSF proteins to reflect AD pathology and cognitive decline. Instead of examining large panels of proteins, our approach focused solely on investigating protein pairs where each selected pair included one protein displaying a strong association with CSF amyloid beta peptides and the other with CSF t-tau and p-tau. This allowed us to identify protein combinations that are not only informative but also interpretable and translatable into clinical practice. The significance of this research is enhanced by the growing number of new AD disease-modifying treatments entering clinical trials, emphasizing the need for reliable biomarkers to effectively monitor AD pathology and neurodegeneration and, through that, treatment outcomes.

To form the protein pairs, we considered their associations with CSF AD pathology markers including Aβ40, Aβ42, Aβ42/40, p-tau, t-tau and neuroaxonal damage marker NfL. Through correlation analysis within the A+T+ individuals, we identified two main protein clusters. One cluster contained proteins with no or weak correlation to the CSF markers, but the majority of these proteins showed a high correlation to albumin CSF/serum quotient. Included in this cluster were several proteins with dominant expression in the periphery, such as inter-alpha-trypsin inhibitor heavy chain 1 (ITIH1) and complement component 9 (C9) with main production in liver, kininogen 1 (KNG1) produced mainly in liver and kidney, and apolipoprotein A4 (APOA4) with high expression in the intestine, according to tissue expression analysis in the Human Protein Atlas. CSF levels of these proteins have previously been shown to be strongly associated with blood-brain barrier integrity [28]. This implies that proteins from this cluster originate from non-CNS tissues or have a mixed origin in the brain and other tissues. The second cluster consisted of proteins with a weak correlation to the albumin quotient but a strong correlation to the CSF AD markers, suggesting mainly CNS origin. This cluster was further divided into two subclusters. One subcluster contained proteins that mainly correlated with amyloid CSF markers Aβ40 and Aβ42, and the second subcluster comprised proteins mainly correlated with CSF p-tau and t-tau. Interestingly, protein pairs with one protein from each of these two subclusters showed high accuracy in separating A+T+ individuals from A-T- individuals with SCD compared to individual proteins (AUC = 0.98 for GAP43 + PTPRN2, compared to AUC = 0.81 for GAP43 alone). The best separation was observed for protein pairs including GAP43, SNCB, NRGN or AMPH from the tau-associated subcluster. These four proteins show enriched expression in the brain according to the Human Protein Atlas and are located at the synapse. Their increased CSF levels may reflect synaptic loss or dysfunction in the brain, which correlates with the severity of AD [8, 29]. Only weak correlations were observed between these proteins and NfL, which is in accordance with previous studies suggesting that NfL and synaptic markers reflect different neurodegenerative processes [12, 30]. According to Wellington et. al, the increased levels of NRGN in CSF are specific to AD pathology and do not occur in other neurodegenerative disorders, which is also in accordance with its strong correlation to t-tau and p-tau [31]. However, Willemse *et. al* contradict this finding, suggesting NRGN to be a disease-non-specific marker for synaptic degeneration [14]. GAP43, SNCB and AMPH are all pre-synaptic proteins and have also previously been associated with AD [12, 15–19]. Although to our knowledge, neither these proteins nor NRGN have been previously studied in pairs with other CSF proteins. In this study, we demonstrate that their value as AD biomarkers substantially increases when used in ratio with amyloid-correlated proteins, such as PTPRN2. We hypothesise that using proteins in pairs has an additive effect and simultaneously adjusts for general variations in CSF protein levels between individuals, thereby enhancing the differences in protein levels resulting from the pathology. We assume this explanation based on the observation that although the span of the protein levels is wide between individuals, the majority of the proteins from the brain-derived subcluster correlate well with each other within an individual, suggesting consistent relative protein levels within individuals. In A-T- subjects, proteins from both subclusters also correlate well with both amyloid and tau CSF markers. In subjects with AD pathology, these correlations are disrupted. While some proteins increase together with p-tau and t-tau (such as GAP43), other proteins keep their levels more stable independently of the pathology, similarly to Aβ40, or potentially decrease with the pathology, similarly to Aβ42. However, these changes may be relatively small compared to the wide range of inter-individual differences in protein levels, which could limit their clinical utility [32]. This reasoning aligns with the use of the Aβ42/40 ratio which is regarded to adjust for variability that may not be directly related to the underlying amyloid pathology [33]. Similarly, t-tau/Aβ42 and p-tau/Aβ42 ratios have been shown to overperform the individual markers in the prediction of AD pathology [34, 35]. 

Besides increased association with amyloid and tau pathology, we also observed stronger correlations of the protein ratios (amyloid-associated/tau-associated) to cognitive decline compared to protein ratios with both proteins from the same cluster as well as individual proteins, evaluated using five different cognitive scores: MMSE, RAVLT, MoCA, KOD and RCF. Single proteins from the tau-associated cluster were generally weakly negatively correlated to the scores, while the proteins from the amyloid-associated cluster tended to have weak positive correlations. For the tau-associated proteins, negative correlations would be expected as all these proteins are correlated to p-tau and t-tau and show significantly higher CSF levels in A+T+ compared to A-T- individuals with SCD. The positive correlations with amyloid-associated proteins were more unexpected as these do not differ in CSF levels between the sample groups. Several of these proteins were however found to decrease in AD in other studies, such as neuropentraxin 2 (NPTX2) and neuropentraxin receptor (NPTXR) [36, 37]. Similarly, we did not find differences in CSF levels of the nerve growth factor inducible (VGF), which has previously been found decreased in AD as well as other neurodegenerative disorders by others, as summarised by Quinn et. al [38]. In this study, PTPRN2 was one of the proteins with a positive correlation to the cognitive scores but showed no difference when comparing A-T- and A+T+ sample groups. Interestingly, PTPRN2 CSF levels were significantly decreased in individuals with probable AD compared to individuals with SCD and MCI as well in MCI compared to SCD in the discovery cohort when comparing the sample groups based on diagnosis within the amyloid and tau defined sample groups, suggesting association to cognitive impairment independently on AT status. Conversely, PTPRN2 as well as several other proteins from the amyloid-associated cluster including CHL1, amyloid beta precursor like protein 1 (APLP1) or peptidylglycine alpha-amidating monooxygenase (PAM) have previously been identified as BACE1 substrates, suggesting their potential involvement in amyloid processing [39, 40]. More information is needed to understand the involvement of these proteins in AD pathology and neurodegeneration.

There are several limitations to this study that should be taken into consideration. Firstly, in the first parts of the study we employ sample stratification based on CSF amyloid and tau which may produce different results compared to clinical diagnosis stratification. The discovery and validation cohorts employed different diagnostic criteria. In the discovery cohort, the diagnosis relied mainly on clinical assessment. In the validation cohort, the diagnosis was based on both clinical assessment and CSF-based amyloid and tau status and border cases were excluded from the cohort, which might inflate the results observed in the validation cohort. This setting also does not allow investigation of the relation of the measured proteins to diagnosis independently of the AT status in the validation cohort. Five distinct cognitive assessment measures were employed in the discovery cohort. However, their availability varied among individuals, resulting in different sample sizes and group characteristics when comparing the protein patterns to these cognitive scores, potentially introducing limitations in the comparison of associations between the protein levels with the different scores. The consistency of the observations however suggests robustness of the findings. The protein patterns observed in relation to cognition could further be validated only for the MMSE score as no other assessment data were available from the validation cohort. Additionally, this study is cross-sectional and therefore does not enable the assessment of the identified protein pair ratios as biomarkers prospectively. Further assessment using longitudinal samples as well as clinical follow-up is needed to validate their potential to monitor AD pathology and/or cognitive decline over time. Of interest would be also comparison of the protein ratio values with MRI biomarkers which may better reflect the molecular changes associated with neurodegeneration in AD compared to cognitive scores. Lastly, the protein measurements in this study were obtained as relative levels, and therefore direct comparisons between the levels of different proteins cannot be made. The character of relative measurements also does not allow for the establishment of specific cut-offs for the different protein ratios. To do this, new assays measuring the absolute concentrations of the selected proteins in CSF need to be further developed.

## Conclusions

To conclude, our study highlights a significant enhancement in the utility of established biomarkers of synaptic damage, such as GAP43, NRGN, or SNCB, when employed in ratios with correlated brain-derived proteins such as PTPRN2, NCAN or CHL1. We hypothesise that this improvement is attributed to a potential additive effect resulting from the combination of these two components and an effective adjustment for inter-individual variability in cerebrospinal fluid (CSF) protein levels. The herein identified protein pairs demonstrated high accuracy in distinguishing between AD pathology-affected and unaffected individuals, as well as showed strong negative correlations with cognitive decline. While being informative biomarkers, protein ratios are also easily interpretable and their implementation in clinical use might therefore be more feasible compared to larger biomarker panels. With these results, we propose that these protein ratios have the potential to serve as valuable tools for monitoring AD pathology and neurodegeneration, particularly in upcoming clinical trials for novel therapies.

### Supplementary Information

**Additional file 1:** **Supplementary Figure 1*.*** Cohort description based on amyloid and tau CSF levels. Discovery cohort sample classification based on p-tau and Aβ42/40 ratio levels (left); validation cohort sample classification based on p-tau and Aβ42 concentration (right). The dashed lines in both plots mark the cut-off for p-tau (yaxis) and Aβ42/40 or Aβ42 (xaxis) classification.**Supplementary Figure 2*.*** Correlation network of the measured proteins in A+T+ individuals. Only correlations with |rho| > 0.5 are visualised in the network. **Supplementary Figure 3*.*** Correlation network of the measured proteins in A-T- individuals with SCD. Only correlations with |rho| > 0.5 are visualised in the network. **Supplementary Figure 4*.*** Examples of CSF protein level comparison between A-T- (SCD) and A+T+ individuals*.*(A) Proteins with significantly different levels, (B) proteins with no significant differences between the sample groups. **Supplementary Figure 5*.*** Correlation between all measured CSF proteins in A+T+ individuals with SCD*. *The heatmap is clustered based on correlation between the proteins and annotated with the clustering results from Fig. 1A, based on the correlation of the individual proteins to amyloid, tau and NfL markers in A+T+ individuals, and Spearman’s correlation with albumin CSF/serum quotient (Q-Alb)*. ***Supplementary Figure 6*.*** Correlation between all measured CSF proteins in A-T- individuals*. *The heatmap is clustered based on correlation between the proteins and annotated with the clustering results from Fig. 1A, based on the correlation of the individual proteins to amyloid, tau and NfL markers in A+T+ individuals, and Spearman’s correlation with albumin CSF/serum quotient (Q-Alb)*. ***Supplementary Figure 7*.*** Distribution of median ROC AUCs for the protein pairs originating in different clusters in the discovery cohort. The *p*-values were calculated using the Wilcoxon rank sum two-sided test. **Supplementary Figure 8*.*** Association of CSF GAP43 and PTPRN2 to p-tau, Aβ42/40 ratio, age, and sex*. *Scatterplots showing the correlation between CSF levels of GAP43 and PTPRN2, with the individual data points colored by (A) p-tau (on a logarithmic scale), (B) Aβ42/40 ratio, (C) age at sampling, (D) sex together with AT status. Only A-T- (SCD) and A+T+ individuals are included. **Supplementary Figure 9*.*** Distribution of ROC AUCs for the protein pairs originating in different clusters in the validation cohort*. *The *p*-values were calculated using the Wilcoxon rank sum two-sided test. **Supplementary Figure 10*.*** A heatmap of median ROC-AUC values from SVM models from each protein pair in validation cohort. The heatmap is clustered based on the ROC-AUC results and annotated with clustering results from Fig 1A, based on correlation of the individual proteins to amyloid, tau and NfL markers in A+T+ individuals in discovery cohort. **Supplementary Figure 11***.* Comparison of median model AUC values in discovery and in validation cohort. The plot line is set on parameters: intercept = 0, slope = 1. **Supplementary Figure 12*.*** Comparison of correlations of protein pair ratios originating in different clusters to cognitive scores in discovery and validation cohort. The *p*-values were calculated using the Wilcoxon rank sum two-sided test using absolute correlation values. The stars represent the level of significance measured by the *p*-value:  ns> 0.05, * < 0.05; ** < 0.01, *** < 0.001, **** < 1e-04. The color of the stars indicates the cluster with stronger correlations. **Supplementary Figure 13*.*** Comparison of correlations of single proteins or protein pair ratios to cognitive scores between the different protein pair clusters in discovery and validation cohort. The *p*-values were calculated using the Wilcoxon rank sum two-sided test using absolute correlation values. The stars represent the level of significance measured by the *p*-value:  ns> 0.05, * < 0.05; ** < 0.01, *** < 0.001, **** < 1e-04. The color of the stars indicates the cluster with stronger correlations. **Supplementary Figure 14*.*** Correlation between cognitive measurements in discovery cohort. Only A-T- individuals with SCD and A+T+ individuals were included. The correlation between the cognitive scores is evaluated using the Pearson’s method. **Supplementary Table 1*.*** List of analysed proteins and used antibodies. **Supplementary Table 2*.*** Amyloid- and tau- associated proteins and brain-elevation status of their respective genes based on tissue transcriptomic data from the Human Protein Atlas. **Supplementary Table 3*.*** Proteins with significantly different CSF levels in A-T- (SCD) and A+T+ sample groups. **Supplementary Table 4*.*** Median ROC AUC difference between models with only protein pairs as predictors and models with both protein pairs and albumin CSF/serum ratio as predictors in the discovery cohort. **Supplementary Table 5*.*** Pearson correlation between protein pair ratios and cognitive scores in the discovery cohort (top 15). Only A-T- individuals with SCD and A+T+ individuals were included. **Supplementary Table 6*.*** Pearson correlation between protein pair ratios and cognitive scores in the validation cohort (top 15).

## Data Availability

The datasets used and/or analysed during the current study are available from the corresponding author on reasonable request.

## Abbreviations

A+T+/A-T-: Amyloid and tau positive/negative
AD: Alzheimer’s disease
AMPH: Amphiphysin
APLP1: Amyloid beta precursor like protein 1
ARPP21: cAMP-regulated phosphoprotein 21
AUC: Area under the curve
Aβ40: Amyloid beta 40 residues long
Aβ42: Amyloid beta 42 residues long
CHL1: Neural cell adhesion molecule L1-like protein
CI: Confidence interval
CSF: Cerebrospinal fluid
FDA: Food and Drug Administration
GAP43: Growth associated protein 43
KOD: Knowledge of Dementia
MCI: Mild cognitive impairment
MMSE: Mini Mental State Examination
MoCA: Montreal Cognitive Assesment
NCAN: Neurocan
NfL: Neurofilament light chain
NPTXR: Neuropentraxin receptor
NRGN: Neurogranin
p-tau: Phosphorylated tau
PAM: Peptidylglycine alpha-amidating monooxygenase
PET: Positron emission tomography
PTPRN2: Protein tyrosine phosphatase receptor type N2
Q-Alb: Albumin CSF/serum quotient
RAVLT: Rey Auditory Verbal Learning Test
RCF: Rey Complex Figure
ROC: Receiver operating characteristic
SCD: Subjective cognitive decline
SNCB: Synuclein beta
SVM: Support vector machine modelling
t-tau: Total tau
TMEM132D: Transmembrane protein 132D
