# Supplementary material for: CSF protein ratios with enhanced potential to reflect Alzheimer’s disease pathology and neurodegeneration
Source: Mol Neurodegener. 2024 Feb 13;19:15. doi: 10.1186/s13024-024-00705-z (PMC10863228; doi:10.1186/s13024-024-00705-z)
Supplement: Supplementary file 1 — Additional file 1: Supplementary Figure 1. Cohort description based on amyloid and tau CSF levels. Discovery cohort sample classification based on p-tau and Aβ42/40 ratio levels (left); validation cohort sample classification based on p-tau and Aβ42 concentration (right). The dashed lines in both plots mark the cut-off for p-tau (yaxis) and Aβ42/40 or Aβ42 (xaxis) classification.Supplementary Figure 2. Correlation network of the measured proteins in A+T+ individuals. Only correlations with |rho| > 0.5 are visualised in the network. Supplementary Figure 3. Correlation network of the measured proteins in A-T- individuals with SCD. Only correlations with |rho| > 0.5 are visualised in the network. Supplementary Figure 4. Examples of CSF protein level comparison between A-T- (SCD) and A+T+ individuals.(A) Proteins with significantly different levels, (B) proteins with no significant differences between the sample groups. Supplementary Figure 5. Correlation between all measured CSF proteins in A+T+ individuals with SCD. The heatmap is clustered based on correlation between the proteins and annotated with the clustering results from Fig. 1A, based on the correlation of the individual proteins to amyloid, tau and NfL markers in A+T+ individuals, and Spearman’s correlation with albumin CSF/serum quotient (Q-Alb). Supplementary Figure 6. Correlation between all measured CSF proteins in A-T- individuals. The heatmap is clustered based on correlation between the proteins and annotated with the clustering results from Fig. 1A, based on the correlation of the individual proteins to amyloid, tau and NfL markers in A+T+ individuals, and Spearman’s correlation with albumin CSF/serum quotient (Q-Alb). Supplementary Figure 7. Distribution of median ROC AUCs for the protein pairs originating in different clusters in the discovery cohort. The p-values were calculated using the Wilcoxon rank sum two-sided test. Supplementary Figure 8. Association of CSF GAP43 and PTPRN2 to p-tau, Aβ42/40 [file 13024_2024_705_MOESM1_ESM.docx]

**Supplementary figures and tables**

Supplementary figures are listed first, followed by supplementary tables.

**Supplementary figure 1: Cohort description based on amyloid and tau CSF levels.**Discovery cohort sample classification based on p-tau and Aβ42/40 ratio levels (left); validation cohort sample classification based on p-tau and Aβ42 concentration (right). The dashed lines in both plots mark the cut-off for p-tau (y-axis) and Aβ42/40 or Aβ42 (x-axis) classification.


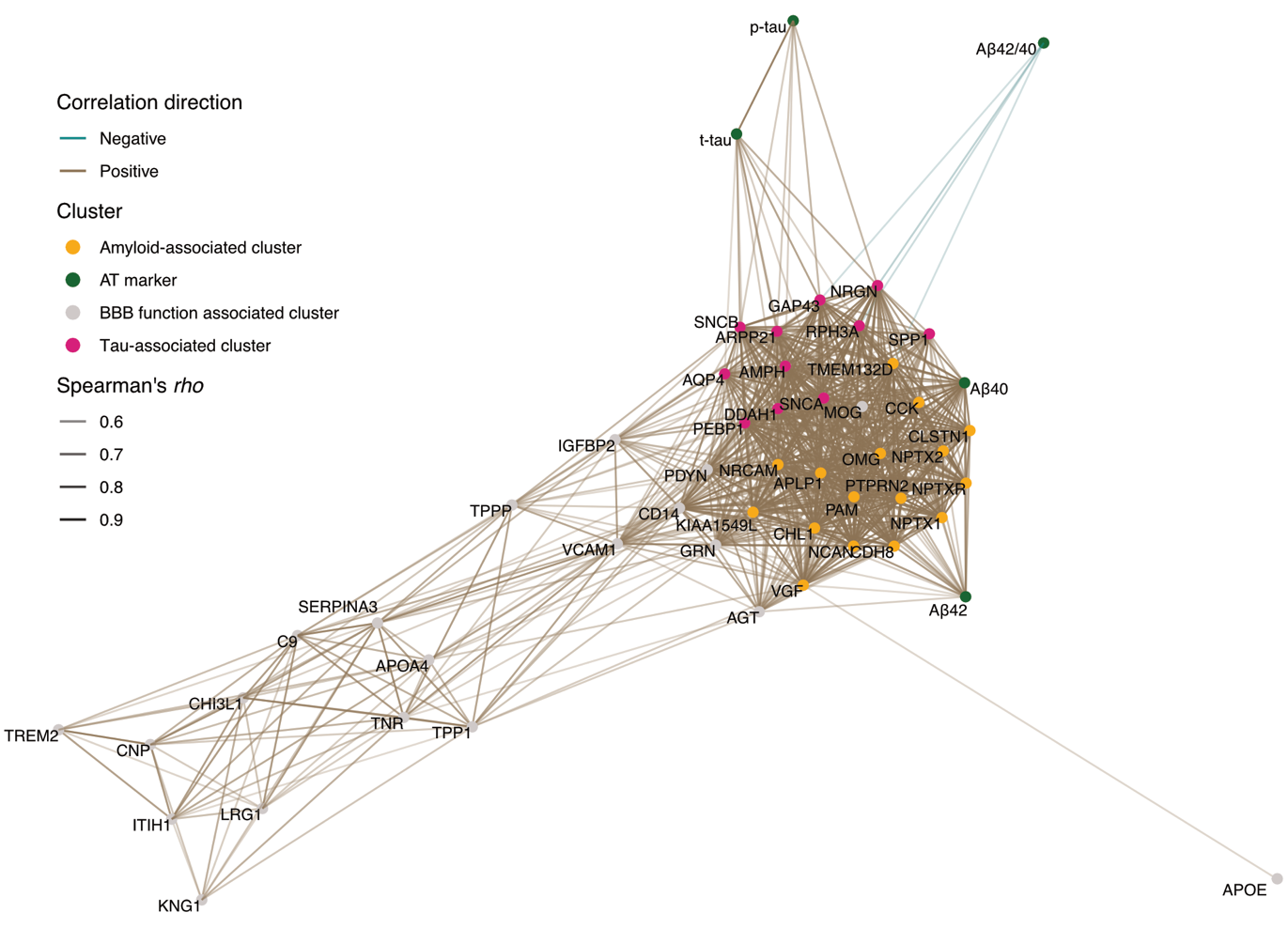


**Supplementary figure 2: Correlation network of the measured proteins in A+T+ individuals.**Only correlations with |rho| > 0.5 are visualised in the network.


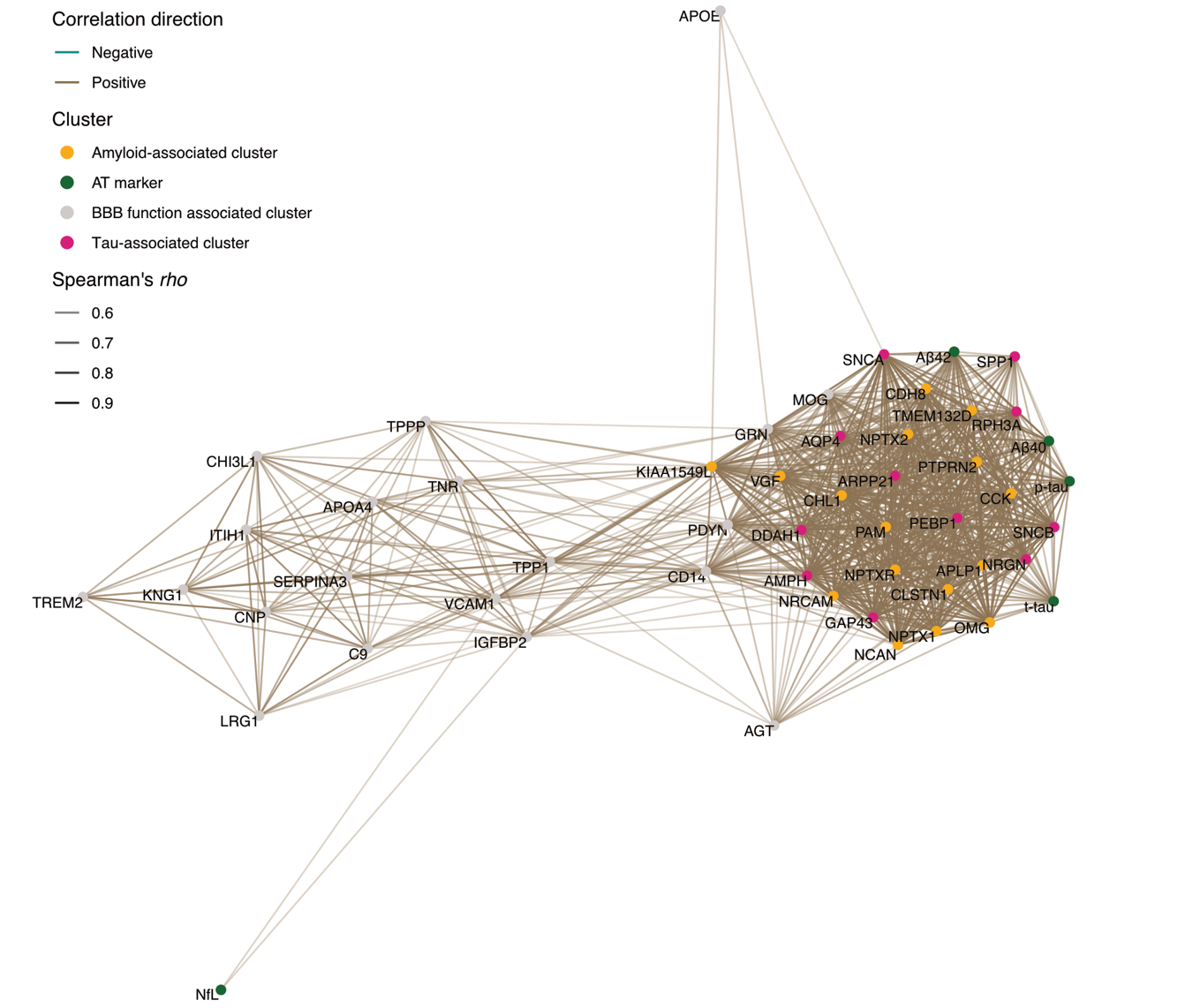


**Supplementary figure 3: Correlation network of the measured proteins in A-T- individuals with SCD.**Only correlations with |rho| > 0.5 are visualised in the network.

**Supplementary figure 4: Examples of CSF protein level comparison between A-T- (SCD) and A+T+ individuals**(A) Proteins with significantly different levels, (B) proteins with no significant differences between the sample groups.

**Supplementary figure 5: Correlation between all measured CSF proteins in A+T+ individuals with SCD**The heatmap is clustered based on correlation between the proteins and annotated with the clustering results from Fig. 1A, based on the correlation of the individual proteins to amyloid, tau and NfL markers in A+T+ individuals, and Spearman’s correlation with albumin CSF/serum quotient (Q-Alb)

**Supplementary figure 6: Correlation between all measured CSF proteins in A-T- individuals**The heatmap is clustered based on correlation between the proteins and annotated with the clustering results from Fig. 1A, based on the correlation of the individual proteins to amyloid, tau and NfL markers in A+T+ individuals, and Spearman’s correlation with albumin CSF/serum quotient (Q-Alb)

**Supplementary figure 7: Distribution of median ROC AUCs for the protein pairs originating in different clusters in the discovery cohort.** The p-values were calculated using the Wilcoxon rank sum two-sided test.

**Supplementary figure 8: Association of CSF GAP43 and PTPRN2 to p-tau, Aβ42/40 ratio, age, and sex**Scatterplots showing the correlation between CSF levels of GAP43 and PTPRN2, with the individual data points colored by (A) p-tau (on a logarithmic scale), (B) Aβ42/40 ratio, (C) age at sampling, (D) sex together with AT status. Only A-T- (SCD) and A+T+ individuals are included.

**Supplementary figure 9: Distribution of ROC AUCs for the protein pairs originating in different clusters in the validation cohort**The p-values were calculated using the Wilcoxon rank sum two-sided test.

**Supplementary figure 10: A heatmap of median ROC-AUC values from SVM models from each protein pair in validation cohort.**
The heatmap is clustered based on the ROC-AUC results and annotated with clustering results from Fig 1A, based on correlation of the individual proteins to amyloid, tau and NfL markers in A+T+ individuals in discovery cohort.


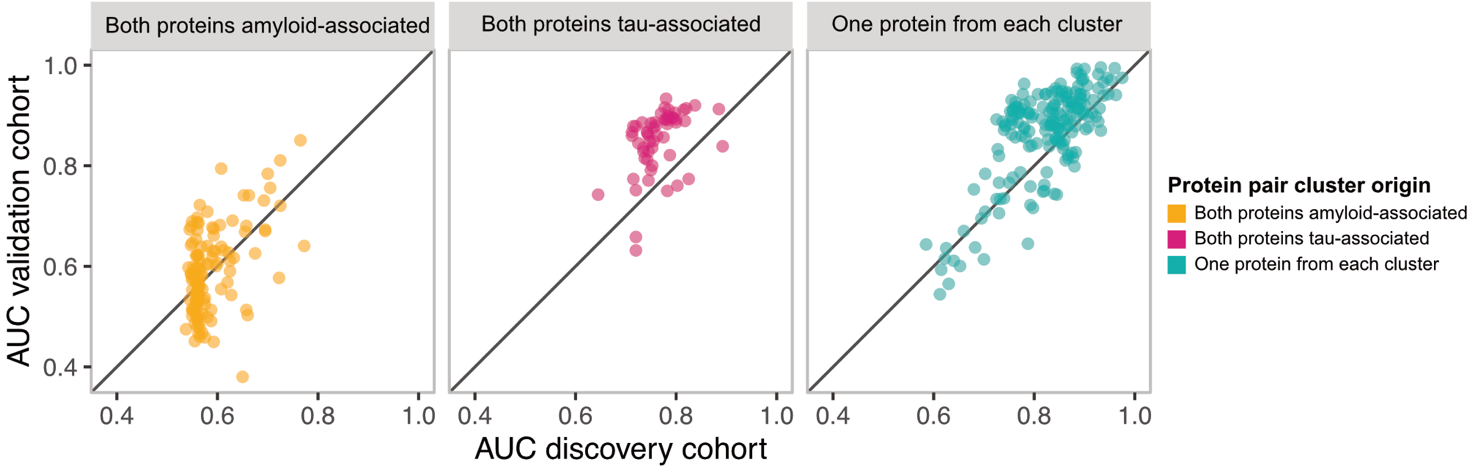


**Supplementary figure 11: Comparison of median model AUC values in discovery and in validation cohort.**The plot line is set on parameters: intercept = 0, slope = 1.

**Supplementary figure 12: Comparison of correlations of protein pair ratios originating in different clusters to cognitive scores in discovery and validation cohort.** The p-values were calculated using the Wilcoxon rank sum two-sided test using absolute correlation values. The stars represent the level of significance measured by the p-value: ns > 0.05, * < 0.05; ** < 0.01, *** < 0.001, **** < 1e-04. The color of the stars indicates the cluster with stronger correlations.

**Supplementary figure 13:** **Comparison of correlations of single proteins or protein pair ratios to cognitive scores between the different protein pair clusters in discovery and validation cohort.** The p-values were calculated using the Wilcoxon rank sum two-sided test using absolute correlation values. The stars represent the level of significance measured by the p-value: ns > 0.05, * < 0.05; ** < 0.01, *** < 0.001, **** < 1e-04. The color of the stars indicates the cluster with stronger correlations.


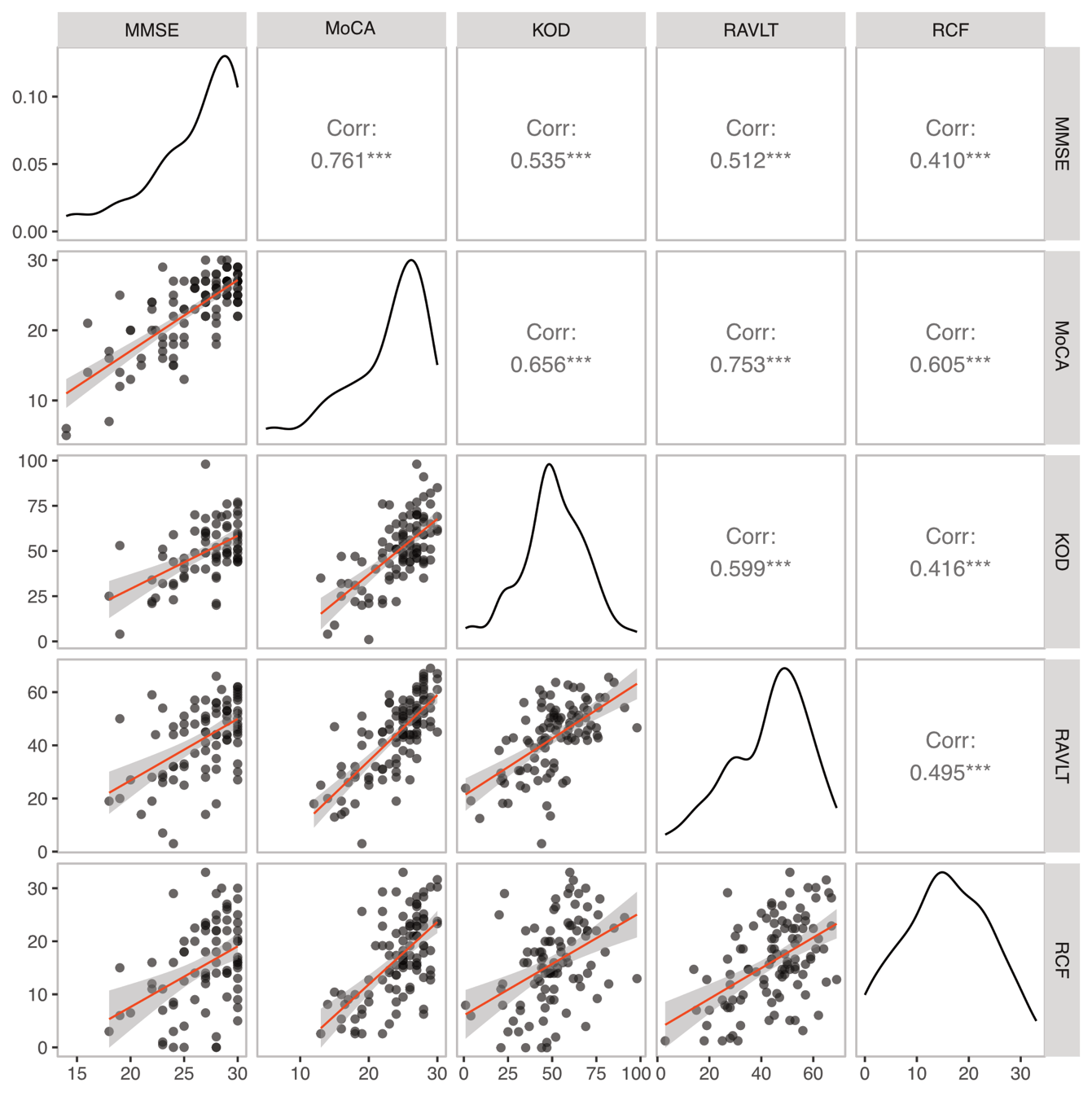


**Supplementary figure 14: Correlation between cognitive measurements in discovery cohort.**Only A-T- individuals with SCD and A+T+ individuals were included. The correlation between the cognitive scores is evaluated using the Pearson’s method.

**Supplementary table 1: List of analysed proteins and used antibodies.**

| Protein name | HGNC ID | Uniprot ID | Antibody |
| --- | --- | --- | --- |
| 2',3'-cyclic nucleotide 3' phosphodiesterase | CNP | P09543 | HPA023278 |
| Amphiphysin | AMPH | P49418 | HPA019829 |
| Amyloid beta precursor like protein 1 | APLP1 | P51693 | HPA028970 |
| Angiotensinogen | AGT | P01019 | AF3156-SP |
| Apolipoprotein A4 | APOA4 | P06727 | HPA002549 |
| Apolipoprotein E | APOE | P02649 | HPA068768 |
| Aquaporin 4 | AQP4 | P55087 | HPA014784 |
| CAMP regulated phosphoprotein 21 | ARPP21 | Q9UBL0 | HPA017303 |
| CD14 molecule | CD14 | P08571 | HPA002035 |
| Cadherin 8 | CDH8 | P55286 | HPA014908 |
| Calsyntenin 1 | CLSTN1 | O94985 | HPA012749 |
| Cell adhesion molecule L1 like | CHL1 | O00533 | HPA003345 |
| Chitinase 1 | CHIT1 | Q13231 | HPA010575 |
| Chitinase 3 like 1 | CHI3L1 | P36222 | HPA072269 |
| Cholecystokinin | CCK | P06307 | HPA069515 |
| Complement C9 | C9 | P02748 | HPA070709 |
| Dimethylarginine dimethylaminohydrolase 1 | DDAH1 | O94760 | HPA006308 |
| Granulin precursor | GRN | P28799 | HPA028747 |
| Growth associated protein 43 | GAP43 | P17677 | HPA013603 |
| Insulin like growth factor binding protein 2 | IGFBP2 | P18065 | HPA077723 |
| Inter-alpha-trypsin inhibitor heavy chain 1 | ITIH1 | P19827 | HPA042049 |
| KIAA1549 like | KIAA1549L | Q6ZVL6 | HPA051594 |
| Kininogen 1 | KNG1 | P01042 | HPA001616 |
| Leucine rich alpha-2-glycoprotein 1 | LRG1 | P02750 | HPA001888 |
| Myelin oligodendrocyte glycoprotein | MOG | Q16653 | HPA021873 |
| Neurocan | NCAN | O14594 | HPA058000 |
| Neurogranin | NRGN | Q92686 | HPA038171 |
| Neuronal cell adhesion molecule | NRCAM | Q92823 | HPA061433 |
| Neuronal pentraxin 1 | NPTX1 | Q15818 | HPA077062 |
| Neuronal pentraxin 2 | NPTX2 | P47972 | HPA058320 |
| Neuronal pentraxin receptor | NPTXR | O95502 | HPA001079 |
| Oligodendrocyte myelin glycoprotein | OMG | P23515 | HPA008206 |
| Peptidylglycine alpha-amidating monooxygenase | PAM | P19021 | HPA042260 |
| Phosphatidylethanolamine binding protein 1 | PEBP1 | P30086 | HPA063904 |
| Prodynorphin | PDYN | P01213 | HPA053342 |
| Protein tyrosine phosphatase receptor type N2 | PTPRN2 | Q92932 | HPA007255 |
| Rabphilin 3A | RPH3A | Q9Y2J0 | HPA002475 |
| Secreted phosphoprotein 1 | SPP1 | P10451 | HPA005562 |
| Secretogranin III | SCG3 | Q8WXD2 | HPA006880 |
| Serpin family A member 3 | SERPINA3 | P01011 | HPA000893 |
| Synuclein alpha | SNCA | P37840 | HPA005459 |
| Synuclein beta | SNCB | Q16143 | HPA035876 |
| Tenascin R | TNR | Q92752 | HPA029859 |
| Transmembrane protein 132D | TMEM132D | Q14C87 | HPA010739 |
| Triggering receptor expressed on myeloid cells 2 | TREM2 | Q9NZC2 | HPA012571 |
| Tripeptidyl peptidase 1 | TPP1 | O14773 | HPA037709 |
| Tubulin polymerization promoting protein | TPPP | O94811 | HPA036575 |
| VGF nerve growth factor inducible | VGF | O15240 | HPA055177 |
| Vascular cell adhesion molecule 1 | VCAM1 | P19320 | HPA069867 |

**Supplementary table 2: Amyloid- and tau- associated proteins and brain-elevation status of their respective genes based on tissue transcriptomic data from the Human Protein Atlas.**

| HGNC ID | Protein name | Cluster | Brain-elevated |
| --- | --- | --- | --- |
| APLP1 | Amyloid beta precursor like protein 1 | amyloid associated proteins | yes |
| CCK | Cholecystokinin | amyloid associated proteins | yes |
| CDH8 | Cadherin 8 | amyloid associated proteins | yes |
| CHL1 | Cell adhesion molecule L1 like | amyloid associated proteins | yes |
| CLSTN1 | Calsyntenin 1 | amyloid associated proteins | low tissue specificity |
| KIAA1549L | KIAA1549 like | amyloid associated proteins | yes |
| NCAN | Neurocan | amyloid associated proteins | yes |
| NPTX1 | Neuronal pentraxin 1 | amyloid associated proteins | yes |
| NPTX2 | Neuronal pentraxin 2 | amyloid associated proteins | no |
| NPTXR | Neuronal pentraxin receptor | amyloid associated proteins | yes |
| NRCAM | Neuronal cell adhesion molecule | amyloid associated proteins | yes |
| OMG | Oligodendrocyte myelin glycoprotein | amyloid associated proteins | yes |
| PAM | Peptidylglycine alpha-amidating monooxygenase | amyloid associated proteins | no |
| PTPRN2 | Protein tyrosine phosphatase receptor type N2 | amyloid associated proteins | yes |
| TMEM132D | Transmembrane protein 132D | amyloid associated proteins | yes |
| VGF | VGF nerve growth factor inducible | amyloid associated proteins | yes |
| AMPH | Amphiphysin | tau associated proteins | yes |
| AQP4 | Aquaporin 4 | tau associated proteins | yes |
| ARPP21 | CAMP regulated phosphoprotein 21 | tau associated proteins | yes |
| DDAH1 | Dimethylarginine dimethylaminohydrolase 1 | tau associated proteins | no |
| GAP43 | Growth associated protein 43 | tau associated proteins | yes |
| NRGN | Neurogranin | tau associated proteins | yes |
| PEBP1 | Phosphatidylethanolamine binding protein 1 | tau associated proteins | no |
| RPH3A | Rabphilin 3A | tau associated proteins | yes |
| SNCA | Synuclein alpha | tau associated proteins | yes |
| SNCB | Synuclein beta | tau associated proteins | yes |
| SPP1 | Secreted phosphoprotein 1 | tau associated proteins | yes |

**Supplementary table 3: Proteins with significantly different CSF levels in A-T- (SCD) and A+T+ sample groups.**

| HGNC ID | Protein name | Uniprot | p-adj | Median fold change | Cluster |
| --- | --- | --- | --- | --- | --- |
| GAP43 | Growth associated protein 43 | P17677 | 5.01E-10 | 1.27 | Tau associated |
| SNCB | Synuclein beta | Q16143 | 5.01E-09 | 1.22 | Tau associated |
| ARPP21 | CAMP regulated phosphoprotein 21 | Q9UBL0 | 5.35E-08 | 1.17 | Tau associated |
| NRGN | Neurogranin | Q92686 | 5.35E-08 | 1.24 | Tau associated |
| AMPH | Amphiphysin | P49418 | 4.69E-07 | 1.21 | Tau associated |
| SPP1 | Secreted phosphoprotein 1 | P10451 | 1.03E-06 | 1.05 | Tau associated |
| AQP4 | Aquaporin 4 | P55087 | 1.82E-06 | 1.20 | Tau associated |
| DDAH1 | Dimethylarginine dimethylaminohydrolase 1 | O94760 | 1.98E-06 | 1.10 | Tau associated |
| PEBP1 | Phosphatidylethanolamine binding protein 1 | P30086 | 8.36E-06 | 1.10 | Tau associated |
| CHIT1 | Chitinase 1 | Q13231 | 5.63E-04 | 1.51 | Plasma/BBB associated |
| APOE | Apolipoprotein E | P02649 | 0.003 | 0.98 | Plasma/BBB associated |
| SNCA | Synuclein alpha | P37840 | 0.003 | 1.11 | Tau associated |
| MOG | Myelin oligodendrocyte glycoprotein | Q16653 | 0.018 | 1.07 | Plasma/BBB associated |
| TPPP | Tubulin polymerization promoting protein | O94811 | 0.034 | 1.02 | Plasma/BBB associated |
| RPH3A | Rabphilin 3A | Q9Y2J0 | 0.039 | 1.11 | Tau associated |

**Supplementary table 4: Median ROC AUC difference between models with only protein pairs as predictors and models with both protein pairs and albumin CSF/serum ratio as predictors in the discovery cohort.**

| tau associated protein | amyloid associated protein | AUC (CI) without Q-Alb | AUC (CI)  with Q-Alb | AUC  difference |
| --- | --- | --- | --- | --- |
| GAP43 | PTPRN2 | 0.98 (0.93–1) | 0.97 (0.91–1) | -0.01 |
| GAP43 | NCAN | 0.96 (0.9–1) | 0.97 (0.91–1) | 0.00 |
| GAP43 | TMEM132D | 0.96 (0.89–1) | 0.95 (0.87–1) | -0.01 |
| GAP43 | CHL1 | 0.95 (0.88–1) | 0.95 (0.88–1) | 0.00 |
| GAP43 | CCK | 0.95 (0.87–1) | 0.94 (0.86–1) | 0.00 |
| GAP43 | NPTX2 | 0.94 (0.84–1) | 0.93 (0.83–0.99) | -0.01 |
| GAP43 | CDH8 | 0.94 (0.84–1) | 0.94 (0.86–1) | 0.00 |
| NRGN | PTPRN2 | 0.94 (0.83–1) | 0.93 (0.83–0.99) | -0.01 |
| SNCB | PTPRN2 | 0.93 (0.84–1) | 0.93 (0.81–1) | -0.01 |
| GAP43 | PAM | 0.93 (0.84–0.99) | 0.92 (0.81–0.99) | -0.01 |
| SNCB | CCK | 0.93 (0.84–0.99) | 0.91 (0.79–1) | -0.02 |
| GAP43 | OMG | 0.93 (0.84–0.99) | 0.92 (0.81–0.99) | -0.01 |
| GAP43 | APLP1 | 0.93 (0.84–0.99) | 0.92 (0.83–0.98) | 0.00 |
| GAP43 | NPTXR | 0.93 (0.84–1) | 0.93 (0.82–0.99) | -0.01 |
| AMPH | PTPRN2 | 0.92 (0.81–0.99) | 0.93 (0.82–1) | 0.01 |
| GAP43 | NRCAM | 0.92 (0.8–0.99) | 0.91 (0.79–0.99) | -0.01 |

**Supplementary table 5: Pearson correlation between protein pair ratios and cognitive scores in the discovery cohort (top 15). Only A-T- individuals with SCD and A+T+ individuals were included.**

| RAVLT | | MoCA | | MMSE | | KOD | | RCF | |
| --- | --- | --- | --- | --- | --- | --- | --- | --- | --- |
| protein pair | cor | protein pair | cor | protein pair | cor | protein pair | cor | protein pair | cor |
| SNCB/PTPRN2 | -0.62 | SNCB/PTPRN2 | -0.61 | SNCB/PTPRN2 | -0.59 | SNCB/PTPRN2 | -0.52 | ARPP21/NPTXR | -0.59 |
| GAP43/PTPRN2 | -0.61 | ARPP21/PAM | -0.59 | SNCB/CHL1 | -0.51 | ARPP21/NCAN | -0.52 | GAP43/CHL1 | -0.59 |
| SNCB/CCK | -0.59 | GAP43/NPTX2 | -0.58 | GAP43/PTPRN2 | -0.5 | ARPP21/VGF | -0.5 | GAP43/NPTX2 | -0.59 |
| GAP43/CCK | -0.57 | ARPP21/PTPRN2 | -0.58 | ARPP21/PTPRN2 | -0.49 | ARPP21/PAM | -0.48 | NRGN/NPTX2 | -0.59 |
| SNCB/NPTX2 | -0.57 | SNCB/NPTX2 | -0.57 | SNCB/CCK | -0.49 | SNCB/VGF | -0.47 | SNCB/NPTXR | -0.58 |
| GAP43/NPTX2 | -0.56 | SNCB/NPTXR | -0.57 | SNCB/TMEM132D | -0.48 | AMPH/PTPRN2 | -0.47 | GAP43/NPTXR | -0.58 |
| GAP43/PAM | -0.56 | GAP43/PTPRN2 | -0.57 | ARPP21/NCAN | -0.47 | SNCB/CHL1 | -0.47 | ARPP21/NPTX2 | -0.58 |
| AMPH/PTPRN2 | -0.56 | SNCB/CCK | -0.56 | SNCB/CDH8 | -0.47 | SNCB/NCAN | -0.47 | SNCB/NPTX2 | -0.58 |
| SNCB/NPTXR | -0.56 | GAP43/CHL1 | -0.55 | SNCB/NCAN | -0.47 | AMPH/CHL1 | -0.46 | PEBP1/NPTXR | -0.57 |
| AMPH/NPTX2 | -0.55 | ARPP21/NPTX2 | -0.55 | GAP43/CHL1 | -0.46 | SNCB/CCK | -0.46 | AMPH/NPTXR | -0.57 |
| SNCB/OMG | -0.55 | NRGN/NPTX2 | -0.55 | GAP43/TMEM132D | -0.46 | NRGN/PTPRN2 | -0.46 | PEBP1/NPTX2 | -0.57 |
| GAP43/CHL1 | -0.54 | SNCB/PAM | -0.55 | NRGN/PTPRN2 | -0.46 | ARPP21/PTPRN2 | -0.46 | AMPH/NPTX2 | -0.57 |
| NRGN/PTPRN2 | -0.54 | GAP43/NPTXR | -0.55 | SNCB/PAM | -0.46 | SNCB/NPTX2 | -0.46 | NRGN/NPTXR | -0.57 |
| ARPP21/PTPRN2 | -0.54 | ARPP21/NCAN | -0.54 | ARPP21/APLP1 | -0.45 | ARPP21/APLP1 | -0.45 | SNCA/NPTXR | -0.56 |
| GAP43/NPTXR | -0.54 | GAP43/PAM | -0.54 | ARPP21/CDH8 | -0.45 | ARPP21/KIAA1549L | -0.45 | SNCA/NPTX2 | -0.56 |

**Supplementary table 6: Pearson correlation between protein pair ratios and cognitive scores in the validation cohort (top 15).**

| MMSE | |
| --- | --- |
| protein pair | cor |
| GAP43/PTPRN2 | -0.77 |
| SNCB/PTPRN2 | -0.76 |
| GAP43/CHL1 | -0.75 |
| GAP43/NCAN | -0.75 |
| GAP43/CDH8 | -0.75 |
| GAP43/PAM | -0.73 |
| NRGN/PTPRN2 | -0.72 |
| SNCB/NCAN | -0.72 |
| NRGN/CDH8 | -0.72 |
| GAP43/VGF | -0.72 |
| AMPH/PTPRN2 | -0.71 |
| SNCB/VGF | -0.71 |
| NRGN/CHL1 | -0.71 |
| SNCB/CHL1 | -0.71 |
| AMPH/CDH8 | -0.71 |
